# Collagen/Glutamate Composite Aerogels with Supramolecular Network Structures Fabricated by Regulating Self-Assembly Behavior for Drug Delivery System

**DOI:** 10.3390/gels11120951

**Published:** 2025-11-26

**Authors:** Chengfei Yue, Ying Yang, Canhui Jiang, Qingyu Wang, Minjie Xu, Liwen Xu, Ming Yang, Min Hu, Ruquan Zhang

**Affiliations:** 1 State Key Laboratory of New Textile Materials and Advanced Processing Technologies, School of Textile Science and Engineering, Wuhan Textile University, Wuhan 430200, China; chengfei.yue@foxmail.com (C.Y.);; 2 Hubei Integrative Technology and Innovation Center for Advanced Fiberous Materials, Wuhan 430200, China

**Keywords:** collagen, glutamate, self-assembly, aerogels, drug delivery, hemostatic capability

## Abstract

Designing and developing multifunctional wound dressings with sustained drug-release capability is a promising strategy for minimizing the risks of wound infection and promoting wound healing. Collagen composite aerogels have been widely employed as a medical device building block, although they still fail to display competitive mechanical properties and sustained drug-release capability. Thus, we solve this challenge by pursuing a multi-scale design method, which utilizes glutamic acid (Glu) to regulate the collagen self-assembly behavior to obtain a network-structured collagen/glutamate composite aerogel with sustained drug release, biocompatibility, and hemostatic ability. Through structural and performance analysis, the Glu endows collagen composite aerogels with excellent structural stability and superior mechanical properties by regulating the intermolecular interaction between collagen molecules, which made the aerogels achieve a supramolecular network structure through the entanglement of high-density collagen fibrils and showed excellent sustained drug-release characteristics. Moreover, collagen/Glu composite aerogels also exhibited outstanding biocompatibility and hemostatic capability. This self-assembly strategy provides new insight aimed at collagen composite aerogels with supramolecular network structures and sustained drug-release capability, making them a promising candidate for wound dressings in future clinical applications.

## 1. Introduction

Drug delivery materials with controlled drug release capability have emerged as a prominent research focus in the field of drug development, offering enhanced stability and improved bioavailability [1,2]. As an advanced porous nanomaterial, aerogel has attracted extensive attention due to its rich surface functionality, large specific surface area, high porosity, tunable network size, and ultra-low density, which also makes it a promising candidate for new drug delivery systems. Aerogel drug delivery materials have more advantages than traditional drug delivery materials [1]. The small molecule drug loading capacity of aerogel drug delivery materials is faster and larger, the restrictions for drugs to enter the internal region of aerogel drug delivery materials are less, and the interaction between drugs and aerogel drug delivery materials is more efficient. More and more research papers on aerogel drug delivery materials reflect the growing interest in this field [3]. However, the application of synthetic polymeric aerogels and inorganic aerogels in the field of drug delivery materials is limited by their poor biodegradability. In contrast, natural biopolymer-based aerogels exhibit excellent biodegradability, bioactivity, and biocompatibility and are increasingly attractive in the research of aerogel drug delivery systems due to their wide availability and flexible preparation [3,4]. Among them, protein-based (silk, collagen, gelatin, and whey) and polysaccharide-based (cellulose, alginate, chitosan, and starch) aerogels are the most typical representatives.

For protein-based aerogels, their unique advantages are becoming more and more popular, including bioactivity, biocompatibility, biodegradability, flexible preparation, and cost-effectiveness. However, despite the increasing importance of protein-based aerogels, there is still a lack of comprehensive information and there remains confusion regarding their application in drug delivery systems [3]. As the most widely distributed and abundant protein in mammals, collagen has been widely utilized in drug delivery systems and surgical sutures [5,6,7,8,9]. The self-assembly of natural collagen is a crucial molecular behavioral feature [10]. The synthesis of collagen fibrils in vivo represents a self-assembly process influenced by cell-mediated biological regulation and the intrinsic properties of collagen molecules. It significantly governs cellular behavior through the maintenance of the extracellular microenvironment [10]. The excellent thermal stability, mechanical properties, and various biological functions of natural collagen fibers rely on their highly organized structure from nanoscale to macroscopic [11]. Hence, referring to the formation process of collagen fibrils in vivo, collagen molecules can provide a controllable alternative to achieve in vitro self-assembly by controlling environmental conditions [11,12,13].

In fact, a large number of studies have confirmed that collagen molecules can self-assemble into ordered aggregates or supramolecular structures in vitro by adjusting environmental conditions, ultimately forming fibrils [10,11,14]. In vitro, collagen molecules can self-assemble into collagen fibrils with typical band-like structures at neutral salt solutions and appropriate temperatures. During this process, collagen molecules can accurately assemble into highly ordered supramolecular structures through the coordinated driving force provided by intermolecular interactions [11,15]. Therefore, understanding the influencing factors of collagen self-assembly in vitro is crucial for designing and preparing collagen composite materials with supramolecular structures. The self-assembly of collagen into gels and scaffolds makes it a suitable material for application at the wound site to protect the injured area. Sargeant et al. [16] designed a novel injectable hydrogel system composed of multi-armed poly(ethylene glycol) and collagen by utilizing the collagen self-assembly behavior. This hybrid hydrogel exhibited physical and biological properties, and tunable mechanical, which makes it suitable for use as an injectable tissue scaffold for the treatment of a variety of tissue defects. Recently, Xing et al. [17] proposed a pathway to design and synthesize collagen hydrogels with adjustable mechanical properties by using gold nanoparticles to trigger the collagen self-assembly behavior. The non-covalent interaction between gold nanoparticles and collagen chains contributes to flow and rapid recovery under applied stress, which makes them ideal gel materials for tissue engineering applications. Milan et al. [18] mixed extracts of mangosteen peel into collagen and chitosan and collagen scaffolds to prepare materials with potential wound dressing applications. Drug release experiments have shown that the release rate of the extract is 30%, indicating that the formulation will not affect this characteristic. It can be seen that collagen-based aerogel material is a favorable matrix for drug delivery materials. The drug release kinetics of collagen aerogels are usually controlled by the collagen matrix characteristics (composition and cross-linking density). However, as a biomaterial, pure collagen has many disadvantages when used directly, such as poor water solubility, low mechanical strength, weak thermal stability, and rapid biodegradation [19,20]. More importantly, traditional collagen material reinforcement methods often focus on the composite with other materials, rarely considering the hierarchical self-assembly behavior of collagen from bottom to top and the interaction between self-assembled bodies, nor achieving mutual adaptation of collagen materials in function and structure [19,21,22,23,24]. Therefore, when designing collagen-based delivery systems, adjusting the self-assembly processing parameters of collagen molecules may be a promising method for constructing collagen delivery systems with tunable physical properties [10].

**Scheme 1:**
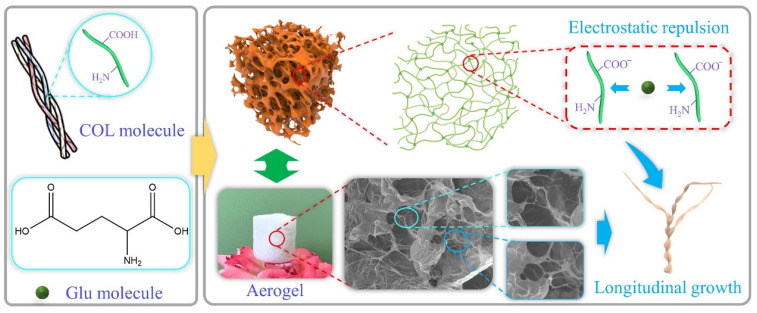
The preparation process for collagen/Glu composite aerogels.

In this work, as shown in Scheme 1, we utilized Glu to regulate the collagen self-assembly behavior and use the self-assembly effect of collagen to design and prepare a collagen/Glu composite aerogel, which had remarkable structural stability, biocompatibility, coagulation properties, and controlled release of model drugs. Firstly, the potential intermolecular interactions between collagen and Glu on the self-assembly behavior of collagen were evaluated using UV-vis and AFM. Subsequently, collagen was dissolved in a certain concentration of Glu solution at 4 °C using continuous mechanical stirring to form a collagen/Glu solution. A simple self-assembly pretreatment process was used to regulate the self-assembly behavior of collagen in the above collagen/Glu solution, obtaining a supramolecular network structure formed by high-density collagen fibril entanglement. Finally, a collagen/Glu composite aerogel was prepared by freeze drying technology. The structures and properties of the collagen/Glu composite aerogels were investigated and discussed using various characterization techniques. These results proved that Glu can endow collagen/Glu composite aerogels with high structural stability and mechanical properties by regulating the intermolecular interaction between collagen molecules. The research results of the drug-release tests in vitro demonstrated that the collagen/Glu composite aerogel has a sustained drug-release characteristic. Moreover, in vitro coagulation test also confirmed the hemostatic ability of collagen/Glu composite aerogels. This work can better understand the structural design and preparation process of collagen based composite aerogels, and provide an effective reference for drug delivery and tissue engineering.

## 2. Results and Discussion

### 2.1. Glu Regulates Collagen Self-Assembly

**Figure 1:**
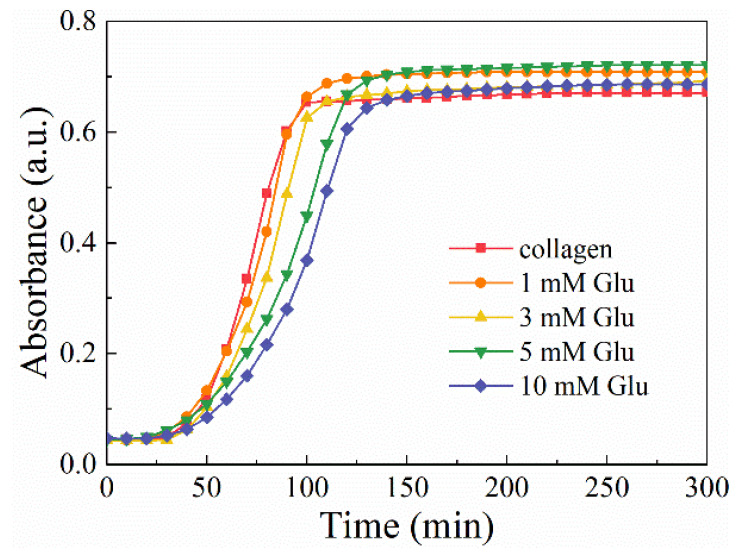
Self-assembly kinetic curves of the collagen/Glu solutions.

To determine the potential mechanism of Glu regulating collagen self-assembly, the turbidity method and AFM measurements were used to investigate its ability to regulate collagen self-assembly behavior and collagen fibril formation with structures similar to natural collagen. Figure 1 shows the self-assembly kinetic curves of the collagen/Glu solutions containing varying Glu concentrations. All turbidity curves of collagen/Glu solutions exhibited a typically S-shaped, consisting of three main periods (lag period, growth period, and plateau period), indicating that collagen molecules in the solution self-assemble to form collagen fibrils under experimental conditions [25,26,27]. Above all, in the absence of Glu, after a simple treatment at 37 °C for about 20 min, collagen fibrils began to form in the pure collagen solution. Then, the rapid self-assembly of collagen molecules further forms a large number of collagen fibrils, causing a rapid increase in turbidity and reaching a plateau at about 100 min. Compared with pure collagen solution, the presence of Glu caused a significant change in the general shape of the turbidity curve of the collagen self-assembly, and the time for growth period troughs to appear was prolonged obviously, demonstrating the role of Glu as an inhibitor of collagen molecule nucleation or growth [28]. Specifically, as the concentration of Glu increases, the lag periods of collagen self-assembly are further prolonged. It should be noted that collagen molecules carry negative charges under neutral conditions, and due to the containment of carboxyl groups, Glu also carries a negative charge. Once Glu was introduced into the collagen solution, the combination of collagen molecules was harder due to electrostatic repulsion, and resulting in delayed lag periods.

### 2.2. Morphologic Analysis of Collagen Self-Assembly Induced by Glu

**Figure 2:**
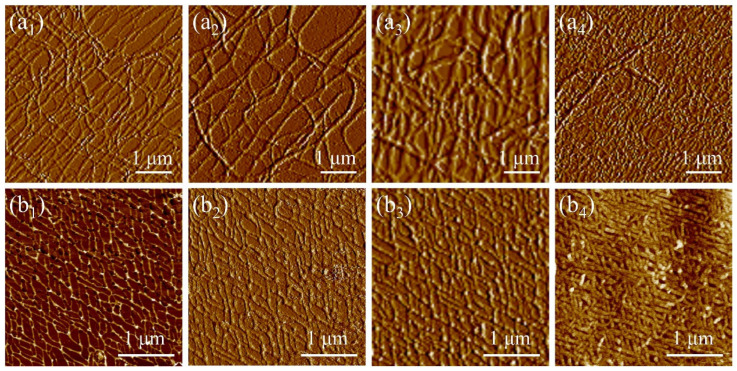
AFM images of collagen fibrils assembled on mica: (**a**) different collagen concentrations (**a_1_**–**a_4_**) 10, 20, 30, and 50 µg/mL); (**b**) 20 µg/mL collagen with different Glu concentrations (**b_1_**–**b_4_**) 0.5, 1.0, 1.5, and 2.0 mM). Scale bar = 1 μm.

To further evaluate the change in collagen self-assembly behavior resulting from the addition of Glu to collagen solution, we characterized the microstructures of self-assembled collagen fibrils using AFM. Figure 2a shows the microstructures of self-assembled collagen fibrils under different collagen concentrations. From Figure 2a_1_, when the collagen concentration was 20 µg/mL, collagen molecules can self-assemble to form oriented parallel microfibrils on the mica surface. Then, the size of collagen microfibrils gradually increased with the increase in collagen concentration (30 µg/mL), forming a network structure on the mica surface (Figure 2a_2_). As shown in Figure 2a_3_, when the collagen concentration increased to 40 µg/mL, the diameter of collagen microfibers reached approximately 200 nm, and a collagen microfibril film also appeared on the mica surface. When the collagen concentration was increased to 50 µg/mL, some collagen microfibrils with crooked morphologies and a few agglomerates were all observed on the previously self-assembled collagen microfibril films. Based on the above, collagen molecules preferred to form consistent and short directionality collagen microfibrils via axial connection in the lower concentrations. The axial connection of single microfibrils will gradually form longer microfibrils with increasing collagen concentration, and the cross-linking between these small microfibrils can even be observed as a dense network structure, ultimately forming a flat collagen film.

The microstructure of self-assembled collagen fibers with different Glu content deposited in Figure 2b can be used to study the effect of Glu on collagen self-assembly behavior. The self-assembly morphologies of collagen fibrils gradually change from a fibrous network structure to a fibrous flat film with increasing Glu concentration. As shown in Figure 2b_1_, under the action of Glu, collagen molecules can self-assemble into a fibrous network structure at a low concentration, which is significantly different from the self-assembly structure of pure collagen mentioned above. With increasing the Glu concentration, the self-assembled structure of collagen fibrils presents a slight gel collagen fibrous film. The self-assembled structure of collagen fibrils gradually showed a slight gel collagen fibrous film with the continuous increase in Glu concentration (Figure 2b_4_). The collagen fibrillogenesis after introducing Glu is controlled to a significant degree by electrostatic interactions, hydrophobic interactions, and hydrogen bonds [29]. Under neutral conditions, negatively charged Glu exhibits electrostatic repulsion with negatively charged collagen, which hinders the free Brownian motion of collagen molecules and slows down their self-assembly behavior. According to the competitive mechanisms of “collagen-collagen” and “collagen-substrate”, the weakening of intermolecular forces between collagen molecules will delay the self-assembly behavior of collagen molecules, causing them to tend to adsorb on the mica surface. This will limit the lateral connection and fusion between collagen fibers, ultimately forming a dense fibrous network structure. The results of AFM analysis indicated that introducing negatively charged Glu in collagen chains plays a remarkable influence on the fibrillogenesis rate, fibril sizes, and micromorphology of the reconstructed collagen fibrils [26,30]. Therefore, based on the turbidity analysis above, the incorporation of negatively charged Glu into collagen under physiological conditions may lead to a conspicuous prolonged lag phase and a distinctively moderative reconstruction rate, but can significantly increase the size or quantity of self-assembled collagen fibrils. However, the negatively charged glutamic acid can promote the self-assembly of collagen building blocks into higher order structures [26]. Overall, the ultrastructure of collagen fibrils under the action of Glu has significant uniqueness, which will provide a theoretical basis for studying collagen materials with different topological structures.

### 2.3. Micromorphology Analysis of Composite Aerogels

**Figure 3:**
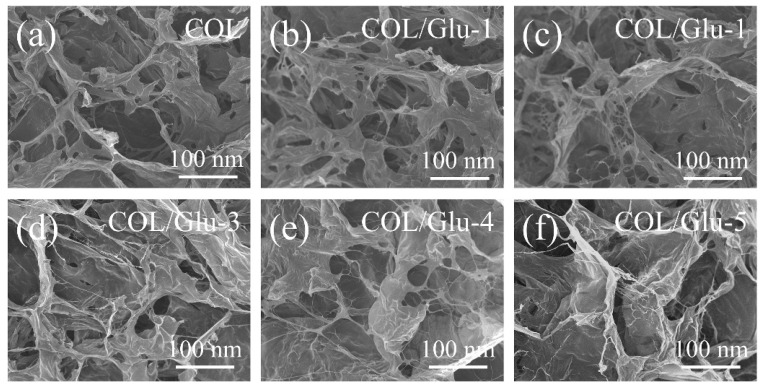
The transverse section micromorphology of collagen composite aerogels: (**a**) COL, (**b**) COL/Glu-1, (**c**) COL/Glu-2, (**d**) COL/Glu-3, (**e**) COL/Glu-4, and (**f**) COL/Glu-5, scale bar = 100 nm.

To investigate the microstructure information of the collagen/Glu composite aerogels, SEM was used to probe the micromorphological information of all relevant samples. Figure 3 shows the transverse section micromorphology of the collagen composite aerogels. All SEM images of the aerogel sample transverse sections indicated a three-dimensional porous network structure [24]. As demonstrated in Figure 3a, the collagen solution can form collagen fibrils after self-assembly pretreatment and reassemble into a three-dimensional monolith to create a porous aerogel. Meanwhile, Glu can influence the collagen self-assembly through electrostatic repulsion, thus making the collagen/Glu composite aerogel produce a unique ultrastructure. The pore size of collagen/Glu composite aerogel decreased with the increase in Glu content. High-density collagen fibril entanglement made the distribution of the supramolecular network structure of collagen composite aerogels more uniform, which ensured the better structural stability of collagen/Glu composite aerogels [31]. Specifically, the collagen solution treated with Glu by a self-assembly pretreatment showed a sharp decrease in pore size and a denser structure. According to the above analysis results and previous reports, the self-assembly behavior of collagen can form a supramolecular network structure through the entanglement of high-density collagen fibrils, which provides a new idea for improving the stability and strength of aerogels [19,20,28]. To further characterize the pore structure of the collagen composite aerogels, the Nitrogen adsorption/desorption isotherms and the corresponding BJH pore size distribution were measured and are illustrated in Figure S1. The total pore volume and mean pore size of mesopores and micropores are illustrated in Table S1. The porosities of COL and COL/Glu-4 were above 90%, and the greater the porosity is, the greater the stress borne by the solid skeleton and the less the stress borne by the liquid in the pores. Meanwhile, the pore diameter calculated by BJH adsorption of S-0 was about 11.27 nm (Table S1). With the introduction of Glu, the electrostatic repulsion makes the COL/Glu composite aerogels produce a unique ultrastructure, and the pore sizes of composite aerogels have decreased, and the distribution of the porous structure is more uniform, which can give composite aerogels better elasticity and is conducive to slowing down drug diffusion and matrix erosion.

### 2.4. Mechanical Properties Analysis

**Figure 4:**
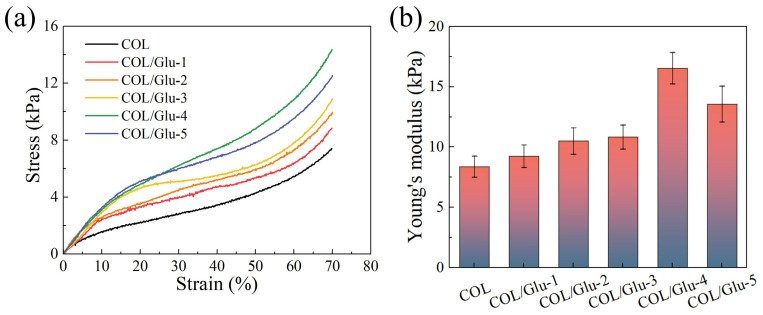
(**a**) compression stress–strain curves and (**b**) Young’s modulus of collagen composite aerogels.

Compression tests were used to investigate the effect of Glu on the structural stability of collagen composite aerogels. As exhibited in Figure 4a, these stress–strain curves of all aerogel samples were consistent with the typical compression behavior of elastic aerogels [32]. Compared with COL, the compressive strength of collagen/Glu composite aerogels is significantly enhanced. According to the above SEM analysis results, the increase in Glu content can promote the formation of collagen fibril network structure, which will be conducive to improving the compressive strength of collagen/Glu composite aerogels [33]. The compressive strength of COL/Glu-4 can reach 14.35 kPa at the Glu concentration of 2.0 mg/mL, which is 1.9 times that of COL. Furthermore, as shown in Figure 4b, the young’s modulus of collagen/Glu composite aerogels also increased first and then decreased with the increase in Glu content, and the Young’s modulus of COL/Glu-4 was the largest (16.55 kPa). The reason for the enhanced compressive strength of COL/Glu-4 was related to the supramolecular network structure based on high-density collagen fiber entanglement, where Glu provides stable electrostatic repulsion between collagen fibers [34]. However, excess Glu possibly increased electrostatic repulsion, causing an increase in the gap in the aerogel, which weakened the structural stability and decreased the compressive strength [35]. Therefore, COL and COL/Glu-4 were selected as the representatives for the subsequent structure and performance studies, considering the analysis results of the microscopic morphology and mechanical properties of collagen composite aerogels.

### 2.5. Structural Properties Analysis

**Figure 5:**
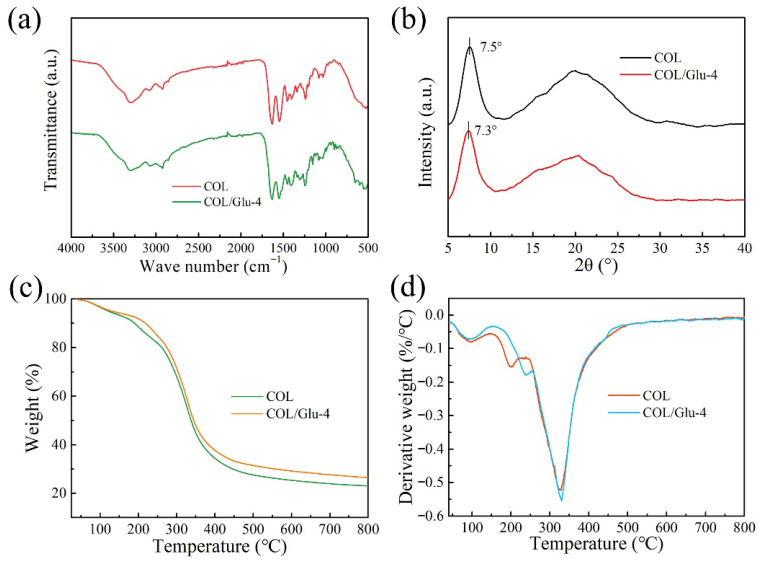
(**a**) FT-IR spectra, (**b**) XRD pattern, (**c**) the TG curves, and (**d**) DTG curves of COL and COL/Glu-4.

FT-IR spectroscopy analysis was used to better understand the interaction between collagen and Glu, and the FT-IR spectra are shown in Figure 5a. The characteristic peaks of COL at 1630, 1544, 1237, and 3294 cm^−1^ corresponded to the C=O stretching vibration (amide I), N–H bending vibration (amide II), C–N stretching vibration (secondary amide), and N–H stretching vibration (amide A), respectively [36]. Due to the high content of COL in the collagen/Glu composite aerogel, the characteristic peak of COL/Glu-4 is roughly the same as that of COL. The possibility of interactions between collagen and Glu in collagen/Glu composite aerogels was studied by analyzing and comparing the changes in absorption intensity and position of amide A, amide I, and amide II bands [37]. The changes in the above characteristic peaks can reflect the formation of intermolecular hydrogen bonds and variations in hydrogen bond strength [38]. Specifically, compared to the spectrum curve of COL, the characteristic peak of the amide A band in COL/Glu-4 shows a significant redshift. The strong hydrogen bonds between collagen fibrils lead to this shift, which affects the mechanical and biological properties of collagen/Glu composite aerogels. These results demonstrated that Glu can enhance the hydrogen bonds between collagen fibrils by regulating collagen self-assembly behavior.

Figure 5b shows the XRD patterns of COL and COL/Glu-4. From Figure 5b, it can be seen that there are two main characteristic diffraction peaks in both the XRD patterns of COL and COL/Glu-4. The XRD pattern of COL has a sharp diffraction peak at 7.5°, which reflects the lateral spacing of collagen molecules [39]. Compared with COL, the diffraction peak of the COL/Glu-4 with added Glu shifted slightly to the left and the intensity also decreased. This is because the electrostatic interaction between Glu and collagen molecules weakens the intermolecular chimera, which slightly increases the chain spacing of collagen fibrils in composite aerogels. Furthermore, the XRD pattern of COL has another broad diffraction peak at 20.0°, which represents diffuse scattering caused by the many structural layers in the collagen fibrils [40]. Compared with the XRD pattern of COL, there is no apparent difference in COL/Glu-4, indicating that the crystalline structure of composite aerogels is stable under the action of Glu. As shown in Figure 5b, the intensity of the relevant diffraction peak of the COL/Glu-4 at 20.0° was slightly enhanced, possibly because Glu could enhance the stability of the supramolecular network structure based on high-density collagen fibril entanglement, which was conducive to enhancing the crystallinity of the composite aerogels. Previous studies have demonstrated that the increase in crystallinity was expected to improve the mechanical strength and modulus of composite aerogels, which is also consistent with the above mechanical property analysis [41].

As illustrated in Figure 5c,d, thermogravimetric analysis (TG and DTG) was employed to examine the thermal characteristics of COL and COL/Glu-4. The TG profiles of COL and COL/Glu-4 displayed three well-defined degradation phases (Figure 5c). For COL, the onset decomposition temperature of the primary degradation phase occurred near 60.0 °C, likely corresponding to the evaporation of adsorbed and structural water molecules. The second decomposition phase commenced at approximately 154.1 °C, indicative of collagen fibril fragmentation from extended polymeric chains to shorter segments. A tertiary degradation stage initiated around 285.3 °C is associated with the progressive breakdown of these truncated chains. The incorporation of Glu notably elevated the thermal degradation thresholds across all stages in COL/Glu-4. The enhanced thermal resistance in COL/Glu-4 principally originates from two synergistic mechanisms: (1) the formation of an intricate supramolecular architecture through dense collagen fibril entanglement, and (2) stabilizing intermolecular forces, including hydrogen bonding between collagen constituents. As exhibited in Figure 5d, DTG analysis corroborates these observations, demonstrating superior thermal endurance in COL/Glu-4 relative to COL. Specifically, Glu integration reinforces the supramolecular network’s structural integrity through enhanced intermolecular interactions, thereby requiring greater thermal energy input for matrix decomposition [41,42].

### 2.6. Drug-Release Behavior Analysis

**Figure 6:**
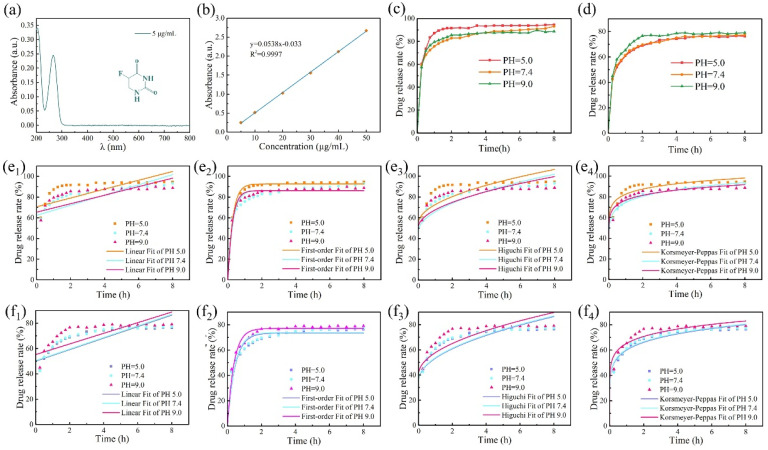
(**a**) UV-vis spectra of 5-FU; (**b**) Standard curve of 5-FU; Drug-release curves of 5-FU from (**c**) COL and (**d**) COL/Glu-4 at different pH. Drug release simulation curves of 5-FU from (**e**) COL and (**f**) COL/Glu-4 at different pH, (**e_1_**) and (**f_1_**) Linear, (**e_2_**) and (**f_2_**) First-order, (**e_3_**) and (**f_3_**) Higuchi, (**e_4_**) and (**f_4_**) Korsmcyer-peppas kinetic models.

As a pyrimidine analog antimetabolite widely employed in oncology therapeutics, 5-Fluorouracil (5-FU) can be easily detected by UV-vis, so it is used as a model drug to evaluate the release behavior of COL and COL/Glu-4. UV-vis spectral analysis (Figure 6a) identified a characteristic absorption peak for 5-FU (20 μg/mL in PBS) at 267 nm, which was subsequently adopted as the analytical wavelength. A linear calibration curve (Figure 6b) was established using Origin 9.0, demonstrating an excellent correlation between absorbance and concentration. Comparative analysis of pH-dependent release kinetics (Figure 6c) revealed rapid equilibrium attainment (~2 h) for COL across all tested pH environments, exhibiting pH-independent burst-release patterns. Conversely, COL/Glu-4 (Figure 6d) demonstrated extended release equilibration (4 h) while maintaining pH insensitivity [43]. This sustained release mechanism originates from Glu-mediated collagen self-assembly, which constructs a dense double-network architecture that entraps 5-FU within interpenetrating supramolecular matrices—a significant advancement over conventional physically blended delivery systems in terms of structural integrity and controlled release performance [44]. Furthermore, according to calculations, the LE of COL/Glu-4 is about 95.3%. Cumulative release curves can provide valuable information for release kinetics and mechanisms [45].

As shown in Figure 6e,f, release kinetics were systematically modeled using different kinetic models (zero-order, first-order, Higuchi, Korsmeyer-Peppas). As detailed in Table S2, neither sample conformed to zero-order kinetics across pH 5.0–9.0. First-order and Korsmeyer-Peppas models showed superior fitting (R^2^ > 0.93), with first-order kinetics providing the closest approximation [46]. This suggests a dual drug distribution mechanism: partial adsorption onto aerogel pore surfaces combined with scaffold matrix incorporation [47]. SEM correlation studies corroborate this model, revealing that the high-density collagen entanglement network creates a supramolecular barrier that restricts drug mobility during hydrogel swelling, thereby enabling controlled 5-FU release [48].

### 2.7. In Vitro Cytotoxicity

**Figure 7:**
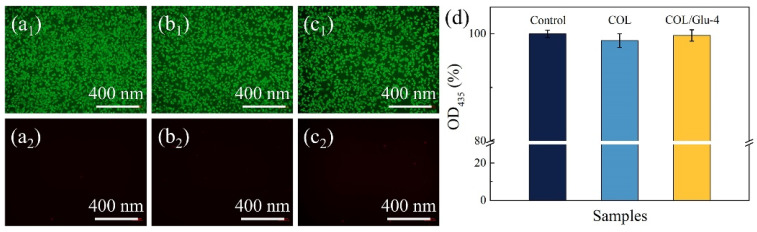
In vitro cytocompatibility analysis of the samples: (**a**–**c**) morphology and quantity of live (green, **a_1_**–**c_1_**) and dead (red, **a_2_**–**c_2_**) cells grown on (**a**) control group, (**b**) COL, and (**c**) COL/Glu-4 for 24 h; (**d**) measurement of L929 cell proliferation by a CCK-8 assay after sample treatment for 24 h. Scale bar = 400 nm.

The biocompatibility assessment of collagen-based biomaterials represents a critical parameter for their biomedical applicability, particularly in drug delivery systems requiring non-cytotoxic carriers. When designing collagen composite aerogels, prioritizing material-cell interface compatibility becomes essential to ensure dermal tissue safety [5,6]. Cellular viability evaluations via CCK-8 assay demonstrated favorable cytocompatibility of both COL and COL/Glu-4 (Figure 7). Fluorescence micrographs (Figure 7a–c) revealed abundant viable L929 cells (green fluorescence) with characteristic spindle morphology and pseudopod extensions across all tested aerogels, confirming non-disruptive cellular interactions. Despite marginally higher proliferation rates in control groups, quantitative analysis (Figure 7d) showed comparable cell viability between COL-3, COL/Glu-4, and literature-reported collagen matrices [20,32]. These collective findings substantiate the low cytotoxicity profile and tissue compatibility of collagen/Glu composite aerogels, fulfilling essential criteria for cutaneous therapeutic applications.

### 2.8. In Vitro Hemostatic and Coagulation Analysis

**Figure 8:**
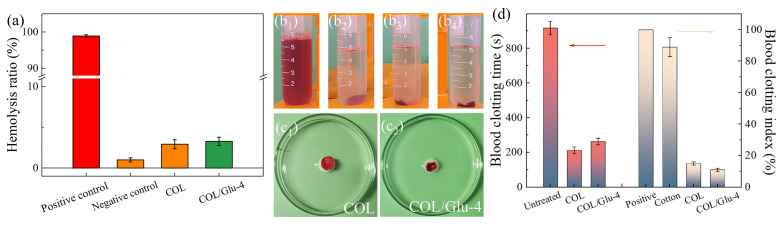
(**a**) Hemolysis ratio of collagen composite aerogels; (**b**) Hemolysis photographs of (**b_1_**) positive control, (**b_2_**) negative control, (**b_3_**) COL, and (**b_4_**) COL/Glu-4; (**c**) Blood clotting photographs of the (**c_1_**) COL and (**c_2_**) COL/Glu-4, (**d**) Blood clotting time and Blood clotting index of the COL and COL/Glu-4.

The primary step in the wound healing process is hemostasis. Therefore, uncontrolled blood coagulation is often the cause of wound dressing failure. First, the blood compatibility of all samples was discussed by performing a hemolysis assay. Figure 8a shows hemolysis photographs of the COL and COL/Glu-4. Deionized water and PBS were tested for the hemolysis assay as positive (significant hemolysis) and negative controls (no hemolysis), respectively. The supernatant was subsequently spectrophotometrically analyzed. The hemolysis ratio is shown in Figure 8b, and it showed the hemolysis ratio of COL and COL/Glu-4 was <5%, which is not significantly different from that of the negative control (PBS group), thus meeting the acceptable standard of hemolysis ratio [49,50]. These results suggest that the collagen/Glu composite aerogels are hemocompatible and can be used as a blood-contacting material. Then, the blood coagulation effect of COL and COL/Glu-4 was explored. As shown in Figure 8c, blood clots formed on the COL and COL/Glu-4 surface, and the color of the rinse water was almost transparent. In contrast, the blood coagulated more completely on the COL/Glu-4. These results showed that the accelerated hemostatic performance of COL/Glu-4 is better than that of COL, providing visual evidence for the rapid hemostatic property of COL/Glu composite aerogel dressings. Subsequently, the hemostatic efficacy of the composite aerogels was analyzed using a blood clotting time (BCT) assay. As illustrated in Figure 8d, the clotting times of COL and COL/Glu-4 were significantly reduced compared to the control group, demonstrating that composite aerogels possess excellent hemostatic properties. The clotting time for COL/Glu-4 was slightly longer compared to COL, which is related to the supramolecular network structure formed by COL fibrils, reducing interactions between COL and blood [50]. The blood clotting index (BCI) represents the number of uncoagulated red blood cells, with a lower BCI indicating stronger hemostatic capability [51]. The experiment used untreated blood as a control, which had a BCI value of 100%. The BCI value for medical cotton balls was 89%, indicating a lower number of uncoagulated red blood cells in the blood that contacted the cotton balls. As shown in Figure 8d, COL/Glu-4 exhibited BCI values below 10%, further confirming the excellent hemostatic performance of composite aerogels.

## 3. Conclusions

In summary, this work has developed a multifunctional aerogel for a drug delivery system based on the self-assembly effect by incorporating Glu into collagen-based composite aerogels and utilizing Glu to regulate the self-assembly behavior of collagen. The multifunctionality of self-assembled collagen/Glu composite aerogel based on the supramolecular network structure of high-density collagen fibril entanglement was evaluated through different structure and performance assays, including structural stability, controlled release of drugs, biocompatibility, and blood coagulation properties. In the first instance, the self-assembly behavior analysis shows that a high-density collagen fibril network can be obtained by regulating the intermolecular interaction between collagen molecules through Glu. The results of the superficial and physicochemical characterization of collagen/Glu composite aerogels indicate that the supramolecular network structure based on high-density collagen fibrils entanglement of the collagen/Glu composite aerogels is formed through the utilization of Glu to regulate the collagen self-assembly behavior. In contrast to COL, Glu could regulate collagen self-assembly behavior, resulting in a relatively higher hydrogen bonding strength and endowing COL/Glu-4 with better structural stability than the other composite aerogels. Mechanical performance analysis shows that the supramolecular network structure of high-density collagen fiber entanglement based on self-assembled aerogels provides sacrificial energy dissipation for enhancing toughness during mechanical damage and obtains excellent compression performance. These findings suggest that the introduction of Glu promotes the rapid formation of this supramolecular network structure based on high-density collagen fibrils in the interior of collagen composite aerogels, which not only improve physical-chemical properties but also enhance thermal and mechanical stability while improving drug-release capabilities in encapsulated 5-FU within composite aerogels. The sustained-release performance of COL/Glu-4 in PBS was mechanistically linked to their dense supramolecular architecture, as evidenced by controlled 5-FU release profiles. First-order kinetics provided an accurate framework for interpreting the diffusion behavior of 5-FU within collagen/Glu composite aerogels, revealing dual drug distribution mechanisms: preferential adsorption onto mesoporous tunnel surfaces and scaffold-matrix integration. Cytocompatibility assessments further confirmed the biological suitability of these aerogels, demonstrating not only excellent cellular viability but also favorable hemocompatibility—critical attributes for therapeutic biomaterial applications. Finally, the collagen/Glu composite aerogel also showed good hemostatic performance in vitro. Based on the above results, the as-prepared collagen/Glu composite aerogel is a suitable alternative to traditional drug delivery materials and opens up a new avenue and an important research basis for the design and synthesis of new wound dressings by regulating collagen self-assembly behavior. It is worth noting that this work lacks in vivo experimental studies, which also indicates the direction for future research.

## 4. Materials and Methods

### 4.1. Materials

Bovine Achilles tendon type I collagen was supplied by Tianjin Sannie Bioengineering Technology Co., Ltd., (Tianjin, China). Glu was purchased from Shanghai Macklin Biochemical Technology Co., Ltd., (Shanghai, China). All of the other reagents were purchased from Tianjin Fengchuan Chemical Reagent Co., Ltd., (Tianjin, China), and they were of analytical grade and used without further purification. Ultrapure water was used for all the experiments in this work.

### 4.2. Preparation of Self-Assembled Collagen Aerogels

A series of Glu solutions (0.5 mg/mL, 1.0 mg/mL, 1.5 mg/mL, 2.0 mg/mL, and 2.5 mg/mL) were prepared by dissolving Glu powder in 0.5 M acetic acid solution and then magnetically starring for 20 min. Subsequently, a series of collagen/Glu solutions were prepared by dissolving a certain amount of collagen in the above Glu solution and then stirring for 8 h with a mechanical stirrer at 4 °C to obtain a uniform and transparent solution with 6.0 mg/mL collagen. The collagen/Glu solution was degassed by freezing centrifugation at 9000 rpm and 4 °C for 20 min before further use. Next, according to previous work, the collagen/Glu solution was transferred to a dedicated mold for pouring and self-assembly pretreatment in a humidity chamber at a temperature of 37 °C and a relative humidity of 50% for 45 min, to induce collagen molecular self-assembly, allowing for sufficient interaction between Glu and collagen molecules [32]. After the cultivation process was completed, the mold containing the aforementioned collagen/Glu solution was then subjected to −80 °C for 12 h for freezing. Finally, the collagen/Glu composite aerogels were obtained by freeze-drying the curing system for 48 h, and the fabrication method for pure collagen aerogels was the same. A series of collagen/Glu composite aerogels with different Glu contents (0.5 mg/mL, 1.0 mg/mL, 1.5 mg/mL, 2.0 mg/mL, and 2.5 mg/mL) were designated as COL, COL/Glu-1, COL/Glu-2, COL/Glu-3, COL/Glu-4, and COL/Glu-5. Without changing the other above-mentioned conditions, 0.3 wt% 5-FU was dissolved in the COL/Glu solution to prepare the drug-loaded composite aerogels.

### 4.3. Characterization

The self-assembly dynamics of collagen solutions with varying Glu concentrations were monitored via ultraviolet-visible spectrophotometry (UV-Vis, TU-1901, Spectrum Analysis Instrument Co., Ltd., Beijing, China). Collagen/Glu solutions were formulated at 4 °C and neutralized to pH 7.4 using 0.1 M NaOH. The collagen/Glu solutions were subsequently incubated at 37 °C under 50% relative humidity to facilitate matrix reconstruction. Aliquots were collected at 10 min intervals, transferred to quartz cuvettes, and analyzed for absorbance at 313 nm.

Atomic force microscopy (AFM, Bruker Icon, Bruker Company, Bremen, Germany) in Smart Scan mode was employed to characterize the morphology of self-assembled collagen fibrils. Collagen solutions prepared using the literature method [25], were stored at 4 °C prior to Glu addition. Collagen/Glu solutions (40 μL) were deposited onto freshly cleaved mica substrates, incubated for 30 min at 4 °C, and further equilibrated for 12 h at 37 °C and 50% relative humidity. After complete desiccation, fibril morphologies were assessed via AFM.

Microstructural features of aerogel samples were evaluated using scanning electron microscopy (SEM, S4800, Hitachi Company, Tokyo, Japan) at an accelerating voltage of 10 kV. Specimens were sputter-coated with gold to minimize charging effects prior to imaging.

The porosities and pore sizes of the aerogels were analyzed by nitrogen adsorption measurements ASAP 2060 (Micromeritics Instrument, Norcross, GA, USA). The aerogels were degassed before the analysis for 10 h at 70 °C, and then they were analyzed with nitrogen at −196 °C. The porosity was calculated as follows:

$$Porosity=\frac{V_{1}\times m_{1}}{V_{0}}\times 100\%,$$

where *V*_0_ is the volume of the sample in its natural state, *V*_1_ is the pore volume of the sample calculated from N_2_ sorption data, and *m*_1_ is the weight of the sample.

Compression tests were conducted on aerogels using an electromechanical testing system (JBDL-50N, MTS Industrial Systems, Shanghai, China) equipped with a 100 N load cell. Samples were compressed to 70% strain at a rate of 10 mm/min, with stress–strain curves derived from five replicate measurements per group.

Fourier-transform infrared spectroscopy (FTIR, Tensor 37, Bruker GmbH, Bremen, Germany) in attenuated total reflectance (ATR) mode was performed to analyze chemical functionalities. Spectra were recorded from 4000 to 400 cm^−1^ at 2 cm^−1^ resolution under ambient conditions.

Crystalline structures of aerogels were determined via X-ray diffraction (XRD, D/MAX-2550, Rigaku Corp., Tokyo, Japan) using Cu/Kα radiation (λ = 0.15418 nm, 40 kV). Diffractograms were collected across a 2θ range of 5–40°.

Thermogravimetric analysis (TGA, STA 449 F3, Netzsch Company, Selb, Germany) assessed the thermal stability of aerogels under nitrogen flow. Samples (~10 mg) were heated from 25 to 800 °C at 10 °C/min.

### 4.4. Drug-Release Analysis of Composite Aerogels

In vitro drug-release kinetics of composite aerogels were evaluated using the following literature methods [20,32,52]. Additional methodological details are provided in the Supporting Information.

As reported in the literature [52], the loading efficiency of COL/Glu-4. A total of 15 mg of COL/Glu-4 was dissolved in 40 mL of PBS. COL/Glu-4 in PBS were moderately shaken on a shaker for 12 h at 40 °C. The above solution was centrifuged at 14,000 rpm to remove the clump of COL/Glu-4. The supernatant was assessed using a UV-vis method, and the absorption band at 267 nm corresponded to 5-FU. The loading efficiency of COL/Glu-4 was determined using the following equations:

$$Loading\;Efficiency\;(LE)=\frac{m_{d}}{m_{1}}\times 100\%$$

where *m*_d_ is the weight of free 5-FU in PBS, *m*_1_ is the weight of initial 5-FU.

### 4.5. In Vitro Cytotoxicity Tests

Cytotoxicity of different aerogel samples was quantified via CCK-8 assays following literature methods [20,32]. Experimental specifics are included in the Supporting Information.

### 4.6. In Vitro Hemostatic and Coagulation Tests

Blood compatibility and coagulation efficacy were investigated through in vitro hemolysis and clotting assays as described previous study [49]. Detailed procedures are provided in the Supporting Information.

## Data Availability

The original contributions presented in this study are included in the article/Supplementary Materials. Further inquiries can be directed to the corresponding authors.

## Supplementary Materials

The following supporting information can be downloaded at: https://www.mdpi.com/article/10.3390/gels11120951/s1, Figure S1: (a) N_2_ adsorption/desorption isotherms and (b) BJH pore size distribution of the COL and COL/Glu-4; Table S1: The surface areas, pore volume, pore diameter and porosities of collagen composite aerogels; Table S2: The correlation coefficient (R^2^) values of samples were calculated by using different kinetic models.
