# Supplementary material for: Collagen/Glutamate Composite Aerogels with Supramolecular Network Structures Fabricated by Regulating Self-Assembly Behavior for Drug Delivery System
Source: Gels. 2025 Nov 26;11(12):951. doi: 10.3390/gels11120951 (PMC12732607; doi:10.3390/gels11120951)
Supplement: Supplementary file 1 [file gels-11-00951-s001.zip › gels-3971565-supplementary.pdf]

Supplementary Materials

Collagen/Glutamate Composite Aerogels with Supramolecular Network Structures Fabricated by Regulating Self-Assembly Behavior for Drug Delivery System

Chengfei Yue [1], Ying Yang [1], Canhui Jiang [1], Qingyu Wang [1], Minjie Xu [1], Liwen Xu [1], Ming Yang [2], Min Hu [1,*] and Ruquan Zhang [1,*]

[1] State Key Laboratory of New Textile Materials and Advanced Processing Technologies, School of Textile Science and Engineering, Wuhan Textile University, Wuhan 430200, China
[2] Hubei Integrative Technology and Innovation Center for Advanced Fiberous Materials, Wuhan 430200, China
* Correspondence: hum@wtu.edu.cn (M.H.); zhangruquan@wtu.edu.cn (R.Z.)

Materials and Methods

Drug release analysis of composite aerogels

The COL and COL/Glu-4 containing 5-FU were dried, and their initial weights were measured. For the drug release behavior study, each specimen was placed in a tube containing 100 mL of the PBS solution. At various time intervals, 4 mL supernatant was taken from the tube and replaced with an equal volume of fresh medium. The amount of 5-FU released from the aerogel materials to the PBS solution was measured using an ultraviolet-visible spectrophotometer (UV, TU-1901, Spectrum Analysis Instrument Co., Ltd., Beijing, China). by measuring the absorbance at 267 nm. A 50 μg/mL stock solution of 5-FU in PBS was used to prepare standard solutions with different concentrations from 0 μg/mL to 50 μg/mL to obtain the calibration curve y = 0.0538x - 0.0024 (x is the absorbance at 267 nm, y is the concentration of 5-FU) and $R^2 = 0.9999$. Drug cumulative release amount was calculated as

$$E_r = \frac{V_e \sum_1^{n-1} C_i + V_0 C_n}{m_{drug}} \times 100\%$$

where E_r (a function of time) is the drug cumulative release amount (%), V_e is the PBS displacement volume (mL), V_0 is the release fluid volume (mL), C_i is release drug concentration at time replacement i.

In vitro cytotoxicity tests

The L929 cells were defrosted and transferred into RPMI-1640 culture medium, and supplemented with 10% fetal calf serum, 100 U/mL of streptomycin, and 100 U/mL of benzylpenicillin. The cells were cultured under a humidified atmosphere containing 5% CO_2 at 37 °C. To analyze the cytotoxicity of the samples, the L929 cells were seeded on different samples and incubated at 37 °C for 24 h. The cell viability of the different samples was measured by a CCK8 assay, and the morphology and quantity of live (Calcein-AM staining, green) and dead (propidium iodide staining, red) cells grown on different samples were observed by a fluorescence microscope. Each group of samples was tested three times to ensure the accuracy of the experimental results.

Hemostatic test and In vitro coagulation tests

The rabbit blood (1 mL) and PBS solution (9 mL, pH 7.4) were added into the centrifuge tube and centrifuged at 1500 rpm for 5 min. The process was repeated 5 times. After centrifugation, pure red blood cells were collected for hemolytic test. Then, pure red blood cells (50 uL) and distilled water (950 L) were taken in the centrifuge tube as positive control group. 10 % red blood cell PBS solution (500 μL) and PBS solution (500 uL) were used as negative control group. The preparation of the different sample groups was the same as that of the negative control group. The different sample groups, positive control group and negative control group were incubated at 37 °C for 2 h. After 24 h, each group was taken out for centrifugation at 2500 rpm for 5 min. Then, each group was taken out and photographed. Finally, the hemolysis rate (HR) of each group was calculated by equation: HR (%) = $[(A_e-A_n) / (A_p-A_n)] \times 100\%$, Where A_e is the absorbance of the sample group, A_p is the positive control group and A_n is the negative control group. For solid materials, hemolysis rate < 5% is qualified.

The aerogel samples were placed on culture dishes. Rabbit blood was dropped on the surface of different samples and incubated at 37 °C for 20 min. After adding 25 mL deionized water, observe the hemolysis of the samples.

Clotting time determination: An in vitro test was conducted to evaluate the clotting time using rabbit blood. In three separate glass vials, 50 mg of aerogel samples were added, respectively. Subsequently, 0.5 mL of rabbit blood was introduced into each vial. These vials were then inverted, and the time required for the blood to cease flowing was measured. Each sample was tested in triplicate, and the average value was recorded.

Clotting index evaluation: The blood clotting index (BCI) was assessed using rabbit blood. SEM was utilized to examine the interaction between composite aerogel surfaces

and red blood cells. Composite aerogels, shaped as cylinders approximately 16 mm in diameter, were positioned in Petri dishes. Rabbit blood was applied to the aerogel surfaces and incubated at 37°C for 20 min. Following incubation, 25 mL of deionized water was added, and the coagulation of blood on the aerogel surfaces was observed. The absorbance of the resulting solution at 540 nm was measured to quantify the concentration of red blood cells. The BCI value was calculated using the following formula:

$$\mathrm{BCI}_{\mathrm{sample}} = \frac{Abs_{sample}}{Abs_{untreated}} \times 100\% \qquad (S1)$$

In the above formula, $Abs_{samples}$ represents the absorbance of each sample, and $Abs_{untreated}$ denotes the absorbance of the blank control group. Each sample was tested in triplicate, and the average value was recorded.

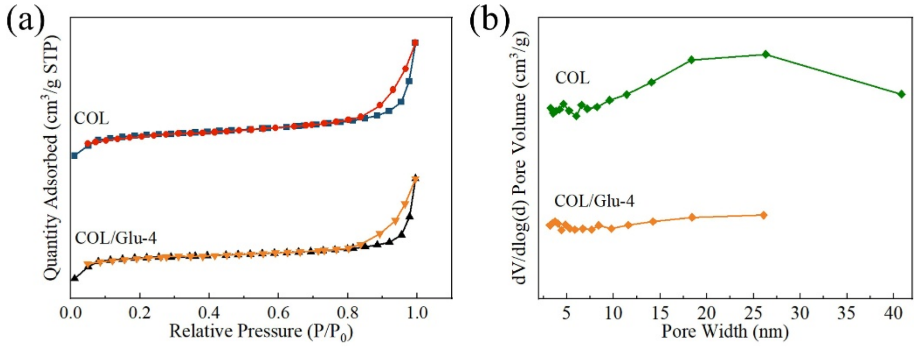

Figure S1. (a) N_2 adsorption/desorption isotherms and (b) BJH pore size distribution of the COL and COL/Glu-4.

Table S1. The surface areas, pore volume, pore diameter and porosities of collagen composite aerogels.

Samples	S_{BET} (m^2 g^{-1}) [a]	V_1 (cm^3 g^{-1}) [b]	PD_{meso} (nm) [c]	Porosities (%)
COL	3.27	14.84	11.27	91.38
COL/Glu-4	4.61	15.18	8.72	94.23

[a] the specific surface area calculated by the BET method; [b] the pore volume calculated by N_2 sorption data; [c] the diameter of mesopores calculated by BJH adsorption.

Table S2. The correlation coefficient (R^2) values of samples were calculated by using different kinetic models.

Kinetic model		Zero-order	First-order	Higuchi	Korsmeyer-Peppas
COL	pH=5.0	0.2280	0.9899	0.4580	0.9545
	pH=7.4	0.3629	0.9364	0.5901	0.9930
	pH=9.0	0.2493	0.9810	0.4800	0.9707
COL/Glu-4	pH=5.0	0.4115	0.9479	0.6585	0.9758
	pH=7.4	0.4117	0.9505	0.6570	0.9768
	pH=9.0	0.3189	0.9726	0.5658	0.9542
